# Human Babesiosis, Maine, USA, 1995–2011 

**DOI:** 10.3201/eid2010.130938

**Published:** 2014-10

**Authors:** Robert P. Smith, Susan P. Elias, Timothy J. Borelli, Bayan Missaghi, Brian J. York, Robert A. Kessler, Charles B. Lubelczyk, Eleanor H. Lacombe, Catherine M. Hayes, Michael S. Coulter, Peter W. Rand

**Affiliations:** Maine Medical Center Research Institute, South Portland, Maine, USA (R.P. Smith Jr,, S.P. Elias, C.B. Lubelczyk, E.H. Lacombe, P.W. Rand);; Maine General Medical Center, Augusta, Maine, USA (T.J. Borelli);; University of Calgary, Department of Medicine, Calgary, Alberta, Canada (B. Missaghi);; Alberta Health Services (Calgary Zone), Calgary (B. Missaghi);; Kadlec Regional Medical Center, Richland, Washington, USA (B.J. York);; University of Maryland, Baltimore, Maryland, USA (R.A. Kessler); University of Vermont College of Medicine, Burlington, Vermont, USA (C. M. Hayes);; Denver Health and Hospital Authority, Denver, Colorado, USA (M.S. Coulter)

**Keywords:** Babesiosis, *Babesia microti*, *Babesia odocoilei*, black-legged tick, *Ixodes scapularis*, Maine, vector-borne infections, deer tick

## Abstract

We observed an increase in the ratio of pathogenic *Babesia microti* to *B. odocoilei* in adult *Ixodes scapularis* ticks in Maine. Risk for babesiosis was associated with adult tick abundance, *Borrelia burgdorferi* infection prevalence, and Lyme disease incidence. Our findings may help track risk and increase the focus on blood supply screening.

Babesiosis caused by *Babesia microti* is a potentially life-threatening parasitic infection transmitted by *Ixodes scapularis*, the deer, or black-legged, tick; it is of increasing concern as a transfusion-acquired illness (*1*,*2*). Since its recognition on Nantucket Island and Cape Cod, Massachusetts, USA, during the 1970s (*3*), human babesiosis from *B. microti* infection has become a public health threat in an increasing number of foci in the northeastern and upper midwestern United States (*1*). Risk for infection by *B. microti* remains geographically more localized than for other pathogens transmitted by *I. scapularis* ticks, such as *Borrelia burgdorferi* (*4*–*6*). This localization may be associated with dense populations of *I. scapularis* ticks and high prevalence of *B. burgdorferi* in ticks (*6*,*7*). Therefore, entomologic data may help predict risk for human babesiosis (*6*).

The presence of *B. microti*–infected ticks in Maine was first documented in 1995 from a town in which Lyme disease was endemic (*7*,*8*). The first case of human babesiosis reported in Maine occurred in 2001, 15 years after the first case of Lyme disease occurred in the state (*8*,*9*). A transfusion-associated case of babesiosis in2007 originated from a blood donor in Maine (*10*). We report on the geographic and temporal expansion of babesiosis in Maine, entomologic correlates of its emergence, and the seroprevalence of *Babesia* spp. in blood donors from the 2 southernmost coastal counties.

## The Study

**Figure 1:**
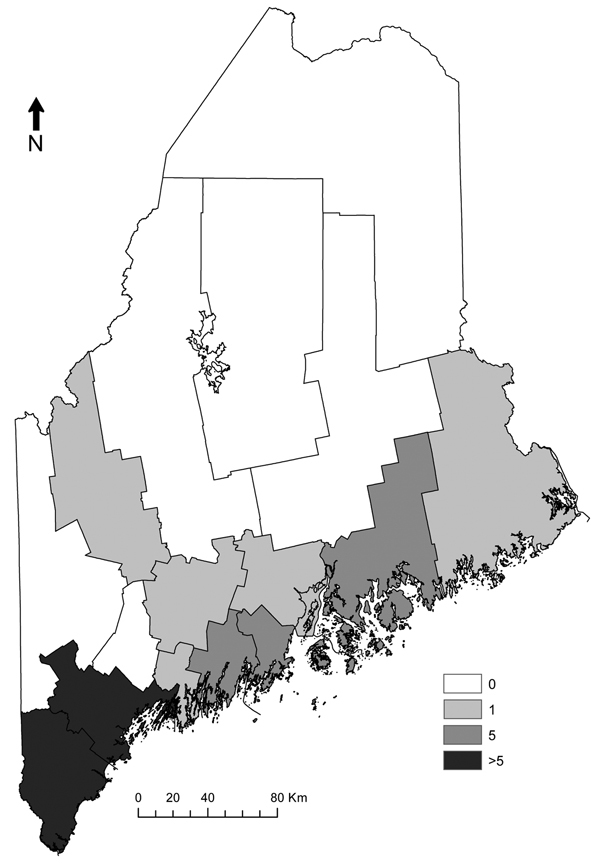
Human babesiosis cases reported by county, Maine, USA, 2001–2011.

We obtained the number of human cases of babesiosis and Lyme disease cases per year and county during1995–2011, from the Maine Center for Disease Control (Table 1) and obtained census data (http://quickfacts.census.gov/qfd/states/23000lk.html) for the years 2000 and 2010. We calculated incidence (cases/100,000 population) using the census for the year 2000 for 1995–2004 and the 2010 census for 2005–2011 (Table 1). Early (2001–2004) babesiosis cases occurred in the 2 southernmost coastal counties, but since 2005, cases have been reported in 8 additional counties, including 2 noncoastal counties (Figure 1). In addition to treatment with antiparasitic drugs, several severely ill patients underwent exchange transfusion. No deaths were reported.

**Table 1:** Prevalence of *Borrelia burgdorferi* in *Ixodes scapularis* ticks and incidence of Lyme disease and human babesiosis, Maine, 1995–2011

Year	Field surveys				Laboratory results					
	No. counties (towns)	No. ticks	No. ticks collected/h		No. ticks positive for *B. burgdorferi*/no. tested (%)	Lyme disease			Babesiosis	
						No. cases*	Incidence		No. cases†	Incidence
1995	5 (6)	498	13		127/308 (41)	45	3.39		0	0
1996	6 (7)	595	12		131/413 (32)	63	4.74		0	0
1997	8 (8)	612	7		162/420 (39)	34	2.56		0	0
1998	3 (7)	580	16		166/399 (42)	78	5.87		0	0
1999	5 (12)	1,444	14		478/886 (54)	89	6.70		0	0
2000	6 (11)	2,390	26		599/1,164 (51)	70	5.27		0	0
2001	5 (7)	967	32		395/779 (51)	108	8.13		1	0.08
2002	3 (5)	773	42		344/669 (51)	218	16.41		2	0.16
2003	5 (9)	986	29		364/758 (48)	175	13.17		3	0.24
2004	4 (9)	799	24		326/688 (47)	224	16.86		5	0.39
2005	5 (8)	1,253	21		197/402 (49)	245	19.23		10	0.78
2006	4 (6)	974	40		342/525 (65)	338	26.53		9	0.71
2007	7 (15)	1,398	22		269/541 (50)	530	41.60		11	0.86
2008	4 (11)	610	34		192/355 (54)	909	71.34		11	0.86
2009	3 (5)	557	34		228/363 (63)	976	76.60		3	0.24
2010	5 (7)	332	14		145/251 (58)	751	58.94		5	0.39
2011	5 (7)	659	32		223/421 (53)	1,007	79.03		9	0.71

*Centers for Disease Control National Notifiable Diseases Surveillance System Lyme disease case definitions: 1995 for 1995, 1996 definition used for 1996–2001, 2011 definition used for 2002–2011. †CDC National Notifiable Diseases Surveillance System 2011 babesiosis case definition.

For the period of 1995–2001, we reviewed published and unpublished data regarding presence of *Babesia* spp. in ticks tested by endpoint PCR (n = 1,433) (*7**–**9*) (Table 2). We examined data from all towns sampled, but to minimize spatial bias, we also examined data only from the town of Wells, from a site sampled in each study. Host-seeking adult ticks made up 66% of the samples, and fed nymphs made up the remainder (*8*) (Table 2). We tested for *Babesia* spp. in ticks using Feulgen stain (*7*–*9*), PCR (*7*–*9*,*11*), or both (Table 2). In Maine, *I. scapularis* ticks are known to harbor 2 *Babesia* spp: *B. odocoilei* (a deer parasite, presumed to be nonpathogenic to humans) and *B. microti* (*7*). However, 18s rRNA sequences (GenBank accession nos. AF028346, AF028343, respectively) to differentiate the species were difficult to obtain because of low DNA concentrations in some samples. Thus, for each study, we calculated the proportion of *B. microti* to *B. odocoilei* as the number of *B. microti*–positive ticks divided by the number of ticks positive for either *B. microti* or *B. odocoilei* (Table 2). Analyzing data from all towns sampled or only Wells, we observed an apparent increase in the ratio of *B. microti* to *B. odocoilei* over time that corresponded with the 2001 appearance of and subsequent increase in reported cases of babesiosis (Table 1). *B. microti* was documented in ticks only from the 2 southernmost counties.

**Table 2:** Emergence of *Babesia microti* in *Ixodes scapularis* ticks, Maine, 1995–2011

Year(s) (ref.)	Sample type	PCR primers†	All towns sampled, N = 90*			Town of Wells	
			PCR	Sequenced		PCR	Sequenced
			No. ticks positive for *Babesia* spp./no. tested	No. *B. microti*/no. sequenced (%)		No. ticks positive for *Babesia* spp./no. tested	No.*B. microti*/no. sequenced (%)
1995–96 (*7*)	Questing adult tick (salivary glands)	PIRO-A/B	28/83	1/25 (4)		11/30	1/10 (10)
1995–1997 (*8*)	Partially engorged nymphal and adult ticks on rodent, dog, cat, and human hosts (salivary glands)	PIRO-A/B	65/455	3/65 (5)		18/148	2/21 (9)
1995–1998 (this study)	Questing adult ticks (salivary glands)	PIRO-A/B	24/208	0/24		8/49	0/8
2003 (*9*)	Questing adult ticks (tick bodies)	PIRO-A/B	15/100	7/15 (47)		15/100	7/15 (47)
2006–07, 2010–11 (this study)	Questing adult ticks (tick bodies)	Bab-1/4 (2006–07), PIRO-A/B	55/728	7/8 (88)		18/126	6/6 (100)

*During 1995–2011, *B. microti* was found only in the southern coastal towns of Kittery, Wells, and Cape Elizabeth; *B. odocoilei* was found in Cape Elizabeth, Wells, and 29 additional, mostly coastal, towns. †The primer pair PIRO-A, PIRO-B targets the 18S rRNA gene, 408 bp for *B. odocoilei*/ 437 bp for *B. microti* (7); the primer pair Bab-1, Bab-4 targets the 18S RNAgene, 238 bp for *Babesia* spp. (*11*).

**Figure 2:**
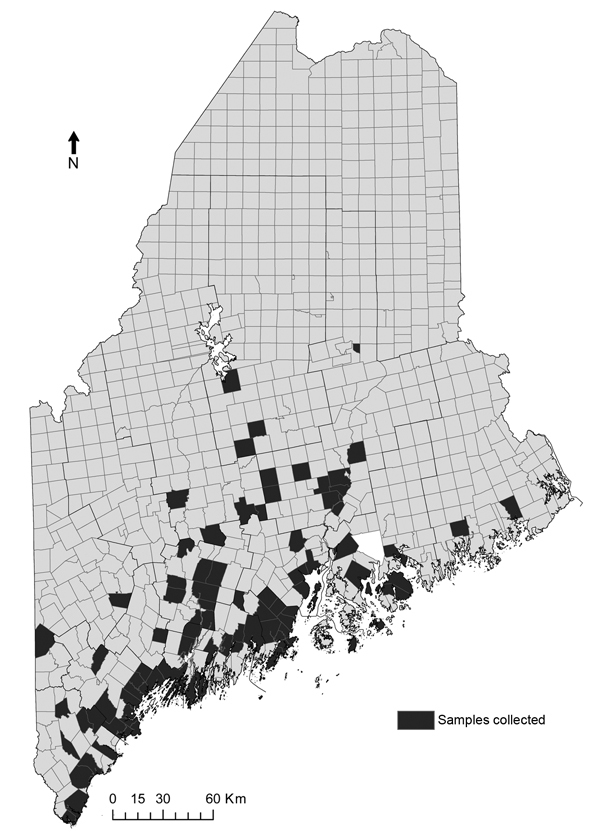
Distribution of towns sampled for questing adult *Ixodes scapularis* ticks, Maine, USA, 1995–2011.

We performed a longitudinal review of the abundance of *I. scapularis* adult ticks and prevalence of *B. burgdorferi* infection. Questing adult *I. scapularis* ticks were collected annually in the fall as previously described (*12*) at 2 long-term study sites in southern coastal Maine and episodically at other sites (Figure 2). A subset of these ticks was tested for *B. burgdorferi* infection by direct fluorescent microscopy (*12*). Table 1 shows upward trends in abundance of adult *I. scapularis* ticks, prevalence of *B. burgdorferi* infection, and incidence of Lyme disease and babesiosis.

Mather et al. (*5*) used logistic regression to demonstrate that abundance of questing nymphal *I. scapularis* ticks (nymphs per hour) of 19–135/hour in wooded areas predicted a ≥20% probability of human babesiosis cases in Rhode Island. Using SAS 9.2 (SAS Institute Inc., Cary, NC, USA), we examined the 20% probability of a babesiosis case as a function of adult ticks collected per hour, *B. burgdorferi* infection prevalence in adult ticks, or Lyme disease incidence. 

We categorized babesiosis cases as present or absent and calculated the number of adult ticks collected per hour, *B. burgdorferi* infection prevalence, and Lyme disease incidence by county and year. We evaluated each univariate model by using the Wald χ^2^_W_ and assuming models to be significant at χ^2^_W_ p≤0.05; and the Hosmer and Lemeshow goodness-of-fit test χ^2^_HL_, assuming suitable fit at χ^2^_HL_ p≥0.10. For the models, all χ^2^_W_≥8.4 and p≤0.004, and all χ^2^_HL_≤11.3 and p≥0.13. A 20% probability of >1 babesiosis case was predicted during years in which abundance of adult ticks collected exceeded 17 per hour (n = 83; 95% CI 12.1%–31.7% for a 20% probability), when *B. burgdorferi* infection prevalence among adult ticks exceeded 34% (n = 74; 95% CI 11.6%–33.6%), or when Lyme disease incidence exceeded 58 cases/100,000 population (n = 272; 95% CI 13.6%–27.9%).

With approval from the Maine Medical Center Institutional Review Board, and in collaboration with Coral Blood Services, Inc., (Scarborough, ME, USA) blood samples from healthy donors were de-identified and screened for *Babesia* antibodies during July–December 2010. Using postal codes, we selected 311 donors from the 2 southernmost Maine counties (Cumberland and York) where *I. scapularis* ticks are more abundant than elsewhere in the state (*13*). Samples were tested via indirect immunofluorescent antibody (IFA) tests as per Krause et al. (*14*) by using serum diluted 1:64. Of 311 blood samples, 10 (3.2%) tested positive for *Babesia* antibody (IgG), which is in the middle of the range for blood donors in the northeastern United States (0.2% in areas where babesiosis is not endemic to 7.3% in highly disease-endemic areas [*15*]). When serum samples were IFA-positive, DNA was extracted from the corresponding whole blood sample at the Yale University School of Public Health laboratory for real-time PCR (Peter Krause, proprietary protocol). *B. microti* DNA was not detected in any of the 10 antibody-positive serum specimens.

## Conclusions

Early studies revealed a higher ratio of presumed nonpathogenic *B. odocolei* to *B. microti* in areas where these species co-exist. The observed temporal shift to *B. microti* in questing adult and fed nymphal *I. scapularis* ticks in this review could be related to ecologic change, or to sampling bias (*7*). Adult *I. scapularis* tick abundance, *B. burgdorferi* infection prevalence among these ticks, and Lyme disease incidence may assist in the prediction of human babesiosis risk. The association observed between number of babesiosis cases and adult *I. scapularis* tick abundance was on a scale similar to that found by Mather et al. (*5*), although that study used nymphal tick abundance. IFA positivity for *Babesia* spp. IgG antibody at >1:64 has a high specificity (90%–100%) (*14*), but its predictive value is uncertain because disease prevalence is unknown for healthy blood donors. As human babesiosis emerges in Maine, refined models correlating babesiosis cases with tick abundance or Lyme incidence, or both, may help track geographic risk and permit targeted screening of the blood supply.

## References

[1] Herwaldt BL, Montgomery S, Woodhall D, Bosserman EA. Babesiosis surveillance—18 states, 2011. MMWR Morbid Mortal Wkly Rep. 2012;61:505–9.>http://www.cdc.gov/mmwr/preview/mmwrhtml/mm6127a2.htm 22785341

[2] Vannier E, Krause PJ. Human babesiosis. N Engl J Med. 2012;366:2397–407 . 10.1056/NEJMra1202018 22716978

[3] Ruebush TK, Juranek DD, Spielman A, Piesman J, Healy GR. Epidemiology of human babesiosis on Nantucket Island. Am J Trop Med Hyg. 1981;30:937–41 http://www.ajtmh.org/content/30/5/937.long. 7283012 10.4269/ajtmh.1981.30.937

[4] Joseph JT, Roy SS, Shams N, Visintainer P, Nadelman RB, Hosur S, Babesiosis in lower Hudson Valley, New York, USA. Emerg Infect Dis. 2011;17:843–7 . 10.3201/eid1705.101334 21529393 PMC3321771

[5] Rodgers SE, Mather TN. *Babesia microti* incidence and *Ixodes scapularis* distribution, Rhode Island, 1998–2004. Emerg Infect Dis. 2007;13:633–5. 10.3201/eid1304.061035 17553286 PMC2726110

[6] Piesman J, Mather TN, Telford SR, Spielman A. Concurrent *Borrelia burgdorferi* and *Babesia microti* infection in nymphal *Ixodes dammini.* J Clin Microbiol. 1986;24:446–7 http://www.ncbi.nlm.nih.gov/pmc/articles/PMC268931/. 3760136 10.1128/jcm.24.3.446-447.1986 PMC268931

[7] Armstrong PM, Katavolos P, Caporale DA, Smith RP, Spielman A, Telford SR. Diversity of *Babesia* infecting deer ticks (*Ixodes dammini*). Am J Trop Med Hyg. 1998;58:739–42 and http://www.ajtmh.org/content/58/6/739.long. 9660456 10.4269/ajtmh.1998.58.739

[8] Holman MS, Caporale DA, Goldberg J, Lacombe E, Lubelczyk C, Rand PW, *Anaplasma phagocytophilum, Babesia microti*, and *Borrelia burgdorferi* in *Ixodes scapularis*, southern coastal Maine. Emerg Infect Dis. 2004;10:744–6 . 10.3201/eid1004.030566 15200875 PMC3323092

[9] Steiner FE, Pinger RR, Vann CN, Grindle N, Civitello D, Clay K, Infection and co-infection rates of *Anaplasma phagocytophilum* variants, *Babesia* spp., *Borrelia burgdorferi*, and the rickettsial endosymbiont in *Ixodes scapularis* (Acari: Ixodidae) from sites in Indiana, Maine, Pennsylvania, and Wisconsin. J Med Entomol. 2008;45:289–97. 10.1603/0022-2585(2008)45[289:IACROA]2.0.CO;2 18402145

[10] Ngo V, Civen R. Babesiosis acquired through blood transfusion, California, USA. Emerg Infect Dis. 2009;15:785–7. 10.3201/eid1505.081562 19402969 PMC2687036

[11] Persing DH, Mathiesen D, Marshall WF, Telford SR, Spielman A, Thomford JW, Detection of *Babesia microti* by polymerase chain reaction. J Clin Microbiol. 1992;30:2097–103 http://www.ncbi.nlm.nih.gov/pmc/articles/PMC265450/. 1500517 10.1128/jcm.30.8.2097-2103.1992 PMC265450

[12] Rand PW, Lubelczyk C, Holman MS, Lacombe EH, Smith RP. Abundance of *Ixodes scapularis* (Acari:Ixodidae) after the complete removal of deer from an isolated offshore island, endemic for Lyme disease. J Med Entomol. 2004;41:779–84 . 10.1603/0022-2585-41.4.779 15311475

[13] Rand PW, Lacombe EH, Dearborn R, Cahill B, Elias S, Lubelczyk CB, Passive surveillance in Maine, an area emergent for tick-borne disease. J Med Entomol. 2007;44:1118–29 . 10.1603/0022-2585(2007)44[1118:PSIMAA]2.0.CO;2 18047214

[14] Krause PJ, Telford SR, Ryan R, Conrad PA, Wilson M, Thomford JW, Diagnosis of babesiosis: evaluation of a serologic test for the detection of *Babesia microti* antibody. J Infect Dis. 1994;169:923–6 . 10.1093/infdis/169.4.923 8133112

[15] Johnson ST, Cable RG, Tonnetti L, Spencer B, Rios J, Leiby DA. Seroprevalence of *Babesia microti* in blood donors from *Babesia*-endemic areas of the northeastern United States: 2000 through 2007. Transfusion. 2009;49:2574–82 . 10.1111/j.1537-2995.2009.02430.x 19821951

